# Accurate comparison of antibody expression levels by reproducible transgene targeting in engineered recombination-competent CHO cells

**DOI:** 10.1007/s00253-014-6011-1

**Published:** 2014-08-27

**Authors:** Patrick Mayrhofer, Bernhard Kratzer, Wolfgang Sommeregger, Willibald Steinfellner, David Reinhart, Alexander Mader, Soeren Turan, Junhua Qiao, Juergen Bode, Renate Kunert

**Affiliations:** 1 Department of Biotechnology/Vienna Institute of BioTechnology (BOKU-VIBT), University of Natural Resources and Life Sciences (Vienna), Muthgasse 18, A-1190 Vienna, Austria; 2 Institute of Immunology, Medical University Vienna, Borschkegasse 8a, A-1090 Vienna, Austria; 3 Department of Genetics, Stanford University School of Medicine, 300 Pasteur Drive, Stanford, 94305 CA USA; 4 WuXi AppTec Co. Ltd, 288 Fute Zhong Road, Shanghai, 200131 China; 5 Institute for Experimental Hematology, Hannover Medical School, Carl-Neuberg-Str.1, D-30625 Hannover, Germany

**Keywords:** Site-directed integration, Cell engineering, Flippase, Specific productivity, RMCE target site

## Abstract

Over the years, Chinese hamster ovary (CHO) cells have emerged as the major host for expressing biotherapeutic proteins. Traditional methods to generate high-producer cell lines rely on random integration(s) of the gene of interest but have thereby left the identification of bottlenecks as a challenging task. For comparison of different producer cell lines derived from various transfections, a system that provides control over transgene expression behavior is highly needed. This motivated us to develop a novel “DUKX-B11 F3/F” cell line to target different single-chain antibody fragments into the same chromosomal target site by recombinase-mediated cassette exchange (RMCE) using the flippase (FLP)/FLP recognition target (FRT) system. The RMCE-competent cell line contains a gfp reporter fused to a positive/negative selection system flanked by heterospecific FRT (F) variants under control of an external CMV promoter, constructed as “promoter trap”. The expression stability and FLP accessibility of the tagged locus was demonstrated by successive rounds of RMCE. As a proof of concept, we performed RMCE using cassettes encoding two different anti-HIV single-chain Fc fragments, 3D6scFv-Fc and 2F5scFv-Fc. Both targeted integrations yielded homogenous cell populations with comparable intracellular product contents and messenger RNA (mRNA) levels but product related differences in specific productivities. These studies confirm the potential of the newly available “DUKX-B11 F3/F” cell line to guide different transgenes into identical transcriptional control regions by RMCE and thereby generate clones with comparable amounts of transgene mRNA. This new host is a prerequisite for cell biology studies of independent transfections and transgenes.

**Electronic supplementary material:**

The online version of this article (doi:10.1007/s00253-014-6011-1) contains supplementary material, which is available to authorized users.

## Introduction

Monoclonal antibodies (mAb) represent the main market fraction of all biotherapeutics with USD 24.6 billion in US sales and a growth of 18.2 % in 2012 (Aggarwal 2014). The main producers for all therapeutic proteins are Chinese hamster ovary (CHO) cells because of their human-like protein expression capabilities, authentic glycosylation patterns, ease of cultivation, and genetic modification (Wurm 2004). Strategies to improve (specific) production titers include genetic engineering of the expression vectors and host cell line combined with optimization of cultivation and feeding strategies (Kim et al. 2012). Cell biological investigations with producer cell lines under different environmental conditions are especially governed by different -omics techniques to refine production capabilities for biopharmaceutical technology (Dietmair et al. 2012; Datta et al. 2013). Although different reviews have been published in detail, so far the success of complex -omics projects is limited (Kantardjieff et al. 2009; Doolan et al. 2013; Clarke et al. 2011). It can be speculated that the cellular model, methodology, or even the bioinformatic input are not yet appropriate to elucidate complex systemic components of cell biology. One challenge when comparing producer cell lines developed from different transfection reactions is to overcome variations in transcription efficacy caused by the “position effect” (Wilson et al. 1990). Together with residual vector-specific components, this effect is a frequent cause of different epigenetic silencing events, among these are histone deacetylation, distinct histone methylation or phosphorylation steps, and DNA-/promoter-methylation patterns (Mutskov and Felsenfeld 2004; Richards and Elgin 2002). These effects may be triggered by integration of the transgene into heterochromatin, leading to the loss or reduction of expression (Kwaks and Otte 2006). Furthermore, it was reported that the transcriptional strength of a promoter is highly dependent on the chromosomal position and the presence of promoter control elements in the surrounding chromatin environment (Nehlsen et al. 2009). Due to these obvious challenges, gene targeting strategies became more and more important for introducing the gene of interest into predetermined chromosomal loci by using site-specific recombinases, such as Cre/loxP (Fukushige and Sauer 1992; Kito et al. 2002) or flippase (FLP)/FLP recognition target (FRT) (O’Gorman et al. 1991; Huang et al. 2007; Turan et al. 2013) systems. The use of two heterospecific, noncompatible FLP recognition target sites in combination with a screening/selection marker enables the isolation of an engineered host cell line containing a chromosomal recombinant cassette in a predefined, transcriptionally active locus. Subsequently, the cassette downstream of the promoter can be replaced by another coding unit based on the same set of flanking heterospecific FRT sites and free of nonessential vector components (“recombinase-mediated cassette exchange” (RMCE)) exploiting the potential of yeast FLP recombinase (Qiao et al. 2009). Our contribution describes the generation of a new host cell line capable to integrate different transgenes into the same chromosomal locus driven by the same transcriptional control elements. Our new CHO host cell line DUKX-B11 F3/F was developed by random integration of a RMCE-competent cassette followed by target site selections to guarantee a homogenous cell population with an expedient cassette exchange and transcription potential. We verified the potential of the new host cell line by the expression of two model proteins, the antibody fragments 3D6scFv-Fc and 2F5scFv-Fc, at the established RMCE target sites. The clones were grown in T25 and spinner flasks for routine cultivation or batch cultivation experiments to analyze specific transcript- and product-formation capabilities. The established DUKX-B11 F3/F cell line provides a stable, retargetable chromosomal locus, indicated by homogenous intracellular green fluorescent protein (gfp) expression, capable to exchange different transgenes into the same transcription control region by site-specific recombination. After selection, scFv-Fc clones developed by RMCE showed reproducible transcription and secretion levels demonstrating the suitability of DUKX-B11 F3/F for comparison of different recombinant producer cell lines.

## Materials and methods

### Plasmids

The cytomegalovirus (CMV) promoter sequence-coding vector pCMV-F3-gfp-F was used for random introduction of the initial RMCE-competent cassette as described in Qiao et al. (2009). It contains the CMV promoter sequence driving the expression of the gfp in combination with a HSV thymidine kinase (tk) polyadenylation signal (pA) and two heterospecific FLP recognition target (*FRT*3 and *FRT*) sites downstream of the promoter and 3′ of the pA sequence. The first RMCE-targeting vector pF3-hygromycin phosphotransferase (hyg)/tk-F harbors a fusion protein of hyg/tk and was constructed by PCR amplification of the fusion protein and cloning into *Age*I and *Nhe*I opened pF3-gfp/tk/neomycin phosphotransferase (neo)-F. The second RMCE-targeting vector pF3-gfp/tk/neo-F contains the fusion protein of gfp, tk, and neo (“gtn”) as described in Qiao et al. (2009). The FLP recombinase enzyme was provided by co-transfection of the plasmid pFLPo containing the FLP coding region under control of a phosphoglycerate kinase promoter (Raymond and Soriano 2007) (Addgene, plasmid 13793). Plasmids pF3-3D6scFv-Fc-F and pF3-2F5scFv-Fc-F contained the 3D6scFv (GenBank, CAA001551) or the 2F5scFv (sequence obtained from PDB, 2F5A), respectively, combined by a (GGGGS)_3_ linker and fused to the human IgG1 Fc region (GenBank, CAA49866). 3D6scFv-Fc or 2F5scFv-Fc open reading frames were amplified by PCR and ligated into the *Kpn*I- and *Nhe*I- opened pF3-gfp/tk/neo-F.

### Construction of RMCE host DUKX-B11 F3/F

CHO DUKX-B11 cells (ATCC CRL-9096) (Urlaub and Chasin 1980) were routinely cultivated in suspension under protein- and antibiotic-free conditions in a 125-mL spinner flask (Techne) pregased to 5 % CO_2_ at 37 °C and 50 rpm. DMEM/HAM’s F12 (1:1) medium (Biochrom GmbH) was supplemented with 4 mM l-glutamine (PAA), 0.1 % pluronic-F68 (Sigma-Aldrich), 100 μM hypoxanthine/16 μM thymidine (HT; Invitrogen), 0.25 % soya-peptone/UF (Quest International GmbH), and a protein-free supplement (Polymun scientific). Plasmid pCMV-F3-gfp-F was introduced by nucleofection (Amaxa cell line Nucleofector kit V, Lonza) of 2 × 10^7^ cells using 20 μg of plasmid DNA in ProCHO5 medium (Lonza) supplemented with 4 mM l-glutamine (PAA) and HT (Invitrogen). Twenty-four hours after the first transfection, 1 × 10^6^ cells were co-transfected with 6 μg pF3-hyg/tk-F and 2 μg pFLPo using 80 μg polyethylenimine (PEI; 25 kDa, linear; Polysciences Inc.) to induce the first RMCE reaction. Stable cell pools were selected by limited dilution in a 96-well plate (Nunc) in ProCHO5 supplemented with 4 mM l-glutamine, HT, and 0.2 mg/mL hygromycin B (Invitrogen). GFP-negative and hygromycin-resistant pools were expanded into T25 flasks and used for a second RMCE reaction by co-transfection of 1 × 10^6^ cells with 6 μg pF3-gfp/tk/neo-F and 2 μg pFLPo using 80 μg PEI. G418-resistant cell pools were selected by limited dilution in ProCHO5 supplemented with 4 mM l-glutamine, HT, and 0.5 mg/mL G418 (PAA) followed by expansion into T25 flasks, a second subcloning step and adaptation into 125-mL spinner flasks (Techne).

### Generation and characterization of scFv-Fc producing RMCE subclones CMV-F3-3D6scFv-Fc-F and CMV-F3-2F5scFv-Fc-F

One million cells of RMCE host DUKX-B11 F3/F were co-transfected with 6 μg pF3-3D6scFv-Fc-F or pF3-2F5scFv-Fc-F and 2 μg pFLPo by 80 μg PEI. After 24 h, cells were resuspended in ProCHO5 supplemented with 4 mM l-glutamine, HT, and 1 μM ganciclovir followed by limited dilution in 96-well plates. ScFv-Fc-producing and growing cell pools were selected by enzyme-linked immunosorbent assay (ELISA) and expanded into T25 flasks for analysis of specific growth rates and specific productivities. Two clones of each scFv-Fc variant were chosen for propagation in spinner flasks for routine cultivation by passaging 2 × 10^5^ living cells/mL in a total working volume of 50 mL every 3 to 4 days to keep cells in exponential growth phase. Additional batch experiments in spinner flasks were performed by seeding 2 × 10^5^ living cells/mL in a total working volume of 75 mL in ProCHO5 supplemented with 4 mM l-glutamine and HT without the use of any positive or negative selection. The batch was harvested when the viability dropped below 30 %. Cell number was determined by nuclei preparation with 0.1 M citric acid and 2 % (*w*/*w*) Triton X 100 and particle counting with Coulter counter Z2 (Beckman coulter). Cell viability was determined by 0.5 % trypan blue dye exclusion using a hemocytometer (Neubauer). Product titer in culture supernatant was quantified by sandwich gamma-gamma ELISA. Ninety-six-well Maxisorp plates (Nunc) were precoated with 0.5 μg/mL polyclonal goat anti-human IgG (γ-chain specific) antiserum (Sigma). Standards and samples were quantified in a 2-fold dilution series with goat anti-human IgG-horseradish peroxidase (HRP; γ-chain specific) conjugate (Invitrogen) and stained with orthophenylediamine and H_2_O_2_ (Merck). The resulting color reaction was measured at 492 nm at a reference wavelength of 620 nm on a microplate reader (Tecan). The specific productivity qP was expressed as picograms per cell per day as described in Lattenmayer et al. (2007).

### Measurement of gfp fluorescence and intracellular product accumulation by flow cytometry

Two million cells of DUKX-B11 F3/F were washed and resuspended in PBS for determination of gfp fluorescence. For intracellular scFv-Fc product analysis, 1 × 10^6^ cells were fixed with ice-cold 70 % (*v*/*v*) ethanol and stained with anti-human IgG-R-phycoerythrin (PE) (γ-chain specific) conjugate antibody (Sigma). All flow cytometric analyses were measured on a Gallios flow cytometer (Beckman Coulter). Single cells were gated according to their forward and side scatter properties and analyzed for gfp fluorescence by FL-1 laser channel or for intracellular PE fluorescence by the FL-2 laser channel using Kaluza Analysis Software (Beckman Coulter).

### cDNA preparation and determination of mRNA transcript level by qPCR

Two million cells were used for total RNA isolation using Ambion TRI Reagent Solution (Life Technologies) according to the manufacturer’s protocol. Residual DNA was removed by DNase (Qiagen) treatment in the presence of RNase inhibitor (Life Technologies). Purified RNA was stored at −20 °C in RNase-free water. Complementary DNA (cDNA) was prepared by reverse transcription with M-MLV reverse transcriptase (Promega) using random primers (Promega) according to the manufacturer’s instructions. Concentration and purity was determined with a ND-1000 spectrophotometer (Nano-Drop). Primers for quantitative PCR (qPCR) analysis were designed using the Primer3 Web application (Untergasser et al. 2007) and synthesized by Sigma-Aldrich. A 100-bp amplicon from the Fc part and a 78-bp amplicon of the housekeeping gene β-actin served as an internal control for normalization of the data.

Three-nanogram sample cDNA was denatured for 10 min to reduce broad dispersion of signals as described in Sommeregger et al. (2013) before PCR reaction was started. qPCR analysis including nontemplate and negative controls was performed on the MiniOpticon system (Bio-Rad) using the TaqMan method by denaturation at 95 °C for 5 min followed by 40 cycles at 95 °C for 15 s and 55 °C for 1 min. Fluorescence signal and Cp values were determined by the CFX manager software 2.1 (Bio-Rad) using baseline subtraction and linear regression. The 2^−∆∆Cp^ method (Livak and Schmittgen 2001) was used for relative quantification of messenger RNA (mRNA) expression levels. For statistical significance, three independent biological samples per cell line were analyzed in two technical runs.

## Results

### Establishment and characterization of the RMCE host cell line DUKX-B11 F3/F

**Fig. 1:**
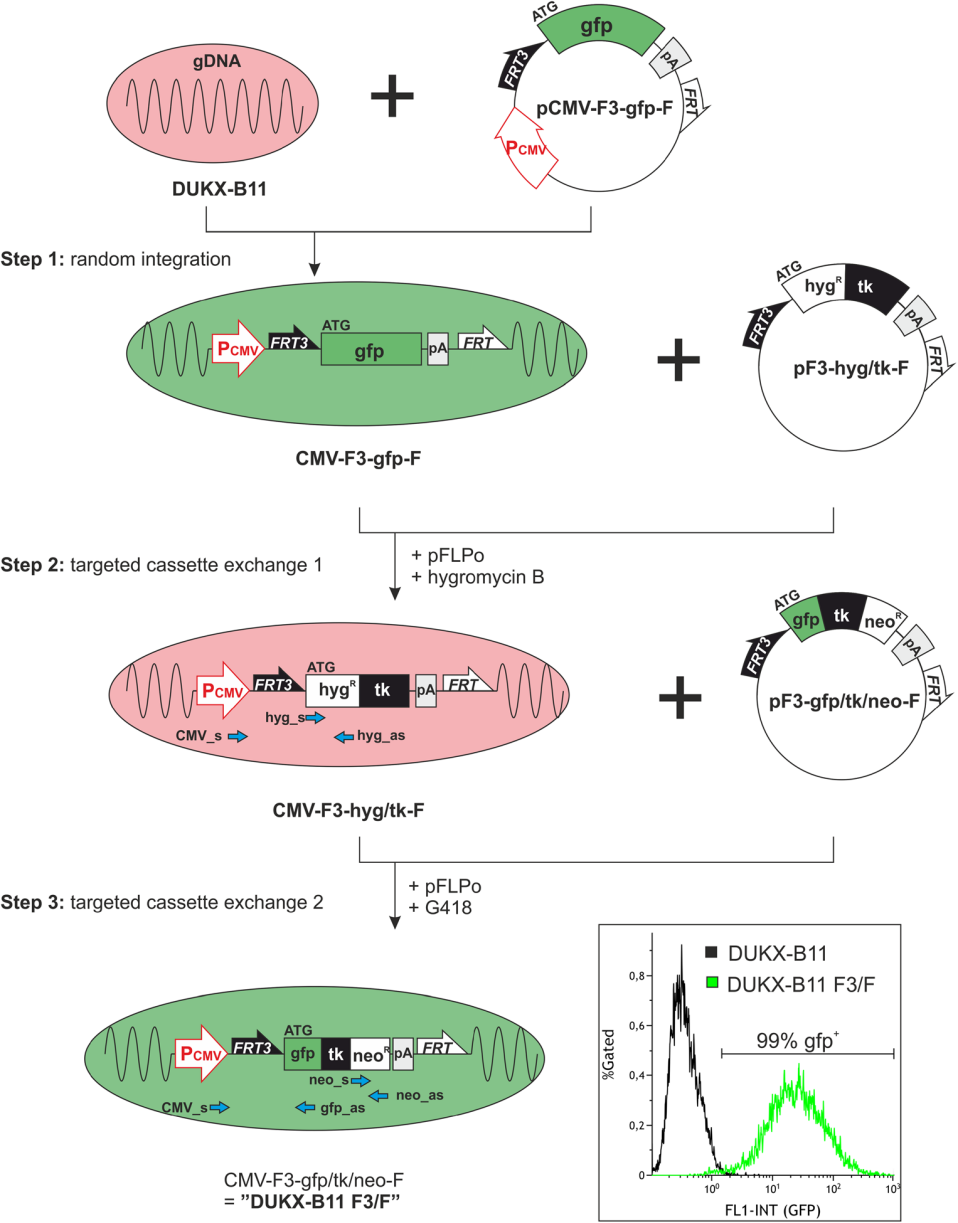
Establishment and homogenous intracellular gfp fluorescence of the RMCE-competent host cell line DUKX-B11 F3/F. DUKX-B11 F3/F was established by random integration of a RMCE cassette on plasmid pCMV-F3-gfp-F (step 1) followed by two consecutive rounds of RMCE (steps 2 and 3) mediated by co-transfection of the targeting vector (pF3-hyg/tk-F or pF3-gfp/tk/neo-F) and the FLP expression plasmid (*pFLPo*). Stable cell clones were selected by hygromycin B (step 2) or G418 (step 3). Primers used for genomic PCR characterization are indicated as *blue arrows*, and results are listed in Table 1 and Online resource 1. The final DUKX-B11 F3/F cell line was analyzed for gfp fluorescence by flow cytometry shown as a single-parameter (*FL1-INT*) histogram. Nonfluorescent DUKX-B11 cells were used as negative control. Plasmids are indicated as *circles*, DUKX-B11 cells are indicated as *pink* or *green ovals*, indicating gfp-negative or gfp-positive fluorescence, respectively, and the cassettes integrated into the host genomic DNA (*gDNA*) are shown. Genetic elements: *pCMV* CMV promoter, *FRT*3/*FRT* spacer-mutant/wild-type FLP recognition target sites, *gfp* green fluorescent protein, *pA* poly A signal, *ATG* start ATG codon, *hygR* hygromycin phosphotransferase, *tk* thymidine kinase, *neoR* neomycin phosphotransferase

Suspension-adapted CHO DUKX-B11 cells were transfected with a plasmid containing the CMV promoter 5′ upstream of a cassette containing the gfp reporter and a polyadenylation signal, flanked by two heterospecific FLP recognition target sites (*FRT*3 and *FRT* ^wt^, abbreviated “F3” or “F”, respectively; step 1 in Fig. 1). The CHO DUKX-B11 cell line was chosen as an RMCE host because of its broad use in industrial biotechnology. For introduction of a first RMCE-competent “donor cassette”, the target unit contained the CMV promoter 5′ upstream of F3 to create a promoter trap in the following transfections. By using an optimized transfection protocol, the genetic expression elements of pCMV-F3-gfp-F were tested in a transient system without positive selection pressure.

The use of a gfp reporter in the first RMCE-targeting construct enables the verification of transfection efficiency. The first RMCE reaction was already initiated 24 h later by co-transfection of the plasmid pF3-hyg/tk-F and the FLP expression plasmid pFLPo to restrict the overgrowth of transfection pools by nonproducers. RMCE donor plasmid pF3-hyg/tk-F comprises a promoterless fusion protein for positive or negative selection by either hygromycin B or ganciclovir, respectively, flanked by the same set of heterospecific *FRT* sites (*FRT3* and *FRT*) (step 2 in Fig. 1). Twenty-four hours after initiation of the first RMCE reaction, the transfection pool was subcloned by limited dilution into 96-well plates in a selection medium containing 200 μg/mL hygromycin B. After 3 weeks, cell growth could be detected in 86 % of the seeded wells by acidification of the phenol red pH indicator. Two out of 576 (0.3 %) totally seeded wells showed a gfp-positive and hygromycin B-resistant cell population that could have been derived from clones containing multiple integrated CMV-F3-gfp-F parental tagging cassettes followed by only partial exchange of some of these targeting cassettes with the F3-hyg/tk-F donor cassettes after the first RMCE reaction. Twelve hyg-resistant clones with gfp-negative phenotype were selected for expansion into T25 flasks. Subclone CMV-F3-hyg/tk-F was selected based on hygromycin B-resistant and gfp fluorescence-negative phenotypes in combination with the highest specific growth rate (data not shown). For evaluation, genomic DNA was extracted and analyzed by PCR (Table 1; Online resource 1) using appropriate primers (blue arrows in Fig. 1). PCR of genomic DNA with primers specific for the CMV promoter and the hyg sequence (CMV_s and hyg_as) generated a specific amplicon, indicating an authentic RMCE reaction with the gfp sequence replaced by the hyg/tk fusion protein. As expected, no amplicon was detected after the first RMCE reaction by PCR using the primer pair specific for the CMV promoter and gfp sequence (CMV_s and gfp_as) confirming the full exchange of parental F3-gfp-F cassettes by RMCE.

**Table 1:** PCR characterization on genomic DNA after first RMCE (clone CMV-F3-hyg/tk-F) and second RMCE (clone DUKX-B11 F3/F) reactions using primers depicted as blue arrows in Fig. 1

Subclone	Primer pair				
	CMV_s gfp_as	hyg_s hyg_as	CMV_s hyg_as	neo_s neo_as	CMV_s neo_as
CMV-F3-hyg/tk-F	−	+	+	−	−
DUKX-B11 F3/F	+	−	−	+	+

Specific PCR amplicons are depicted in Online resource 1

Subclone CMV-F3-hyg/tk-F was subjected to a second round of RMCE to replace the hyg/tk fusion protein with a triple fusion protein consisting of gtn (step 3 in Fig. 1). Again, this exchange cassette did not contain any promoter sequence to make use of the promoter trap to prevent transcription of randomly introduced RMCE donor cassettes. At 24 h posttransfection, stable cell clones were selected by limited dilution in G418-containing media to select subclone CMV-F3-gfp/tk/neo-F based on growth characteristics and gfp fluorescence (data not shown). This final RMCE-competent host cell line, designated “DUKX-B11 F3/F”, was analyzed for an authentic RMCE cassette exchange by PCR analysis of isolated genomic DNA (Table 1; Online resource 1). A specific PCR amplicon could be detected with primers specifically binding to the CMV promoter and gfp reporter sequence (CMV_s and gfp_as). No amplicons were detected with primers specific for hyg (hyg_s and hyg_as). The neo insert was confirmed by using primers binding only to the neo sequence (neo_s and neo_as) as well as primers binding specifically to the CMV promoter sequence and the neo sequence (CMV_s and neo_as). FISH analysis with gtn-specific probes evidenced a single integration site of the RMCE-competent targeting cassette within the DUKX-B11 F3/F host (Online resource 2). Flow cytometry analysis for gfp fluorescence intensities showed that 99 % of DUKX-B11 F3/F cells showed higher gfp fluorescence intensities than the gfp-negative DUKX-B11 host cells (single-parameter FL-1 INT histogram in Fig. 1) with geometric mean, median, and mode values of 22.4, 24.6, and 27.1, respectively, demonstrating the homogenous gfp expression capability of DUKX-B11 F3/F.

### 3D6scFv-Fc and 2F5scFv-Fc product comparison by RMCE

To test its performance as a genomic target site, two anti-HIV single-chain antibodies 2F5scFv-Fc and 3D6scFv-Fc were cloned into a promoterless RMCE vector flanked by the two heterospecific *FRT* sites (pF3-3D6scFv-Fc-F and pF3-2F5scFv-Fc-F) followed by integration into the DUKX-B11 F3/F genome by RMCE equivalent to step 2 or 3 in Fig. 1.

At 24 h posttransfection, stable antibody-producing subclones were selected by limited dilution and negative selection for absence of tk using the deoxyguanosine analog ganciclovir. Twelve clones for each antibody variant were expanded to T25 flasks, and their growth behavior and productivities were measured for ten consecutive passages.

**Fig. 2:**
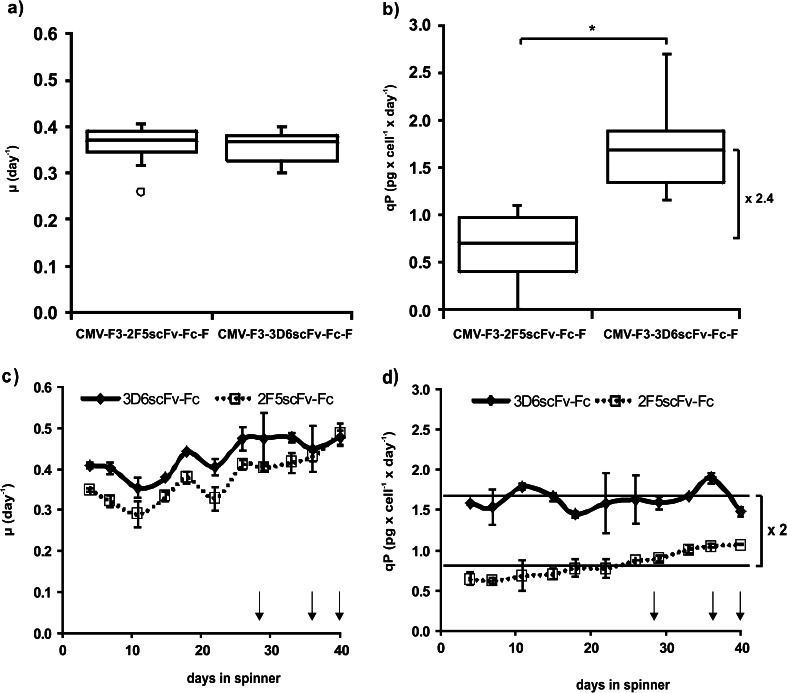
Analysis of specific growth rates and productivities of scFv-Fc producing RMCE cell clones for ten consecutive passages in T25 and routine cultivation in spinner flasks. **a** Specific growth rates μ and **b** specific productivities qP are shown as box plot diagrams of 12 scFv-Fc producing RMCE clones of each antibody variant cultivated for ten consecutive passages in T25 flasks. *Boxes* represent median, first, and third quartiles of 12 clones. Outliers were defined as values ±1.5 × interquartile range (IQR) and are represented as open circles. *Error bars* represent sample maxima and minima within 1.5 × IQR. Factor 2.4 represents difference in median-specific productivities between 3D6scFv-Fc- and 2F5scFv-Fc-producing clones. *p < 0.001—independent two-sample Student’s *t* test. **c** Specific growth rates μ and **d** specific productivities qP of two scFv-Fc-producing clones of each antibody variant are shown as mean values in spinner cultivation for 40 days. *Error bars* represent ±SEM. Days of sampling for flow cytometry and qPCR analyses are shown as *arrows*. Factor 2 represents the mean difference in qP of 3D6scFv-Fc- and 2F5scFv-Fc-producing cell clones

Similar specific growth rates of all selected subclones were confirmed during the T25 cultivation period, suggesting that the different amino acid sequences of the two scFv-Fc variants have no major influence on the cellular metabolism of the established recombinant cell lines (Fig. 2a). The median specific productivity of 12 3D6scFv-Fc-producing subclones was 2.4-fold higher than that of 12 2F5scFv-Fc-producing subclones (Fig. 2b).

Already in course of clone development for 12 antibody-producing subclones of each variant, a highly reproducible and similar cell behavior in terms of specific productivities (qP) and growth rates (μ) could be observed (Fig. 2a, b). By contrast, the development of scFv-Fc-producing clones with conventional random integration of plasmids resulted in much higher variability in specific productivities and growth rates between individual subclones producing the same antibody variant, indicated by a higher interquartile range (midspread) in Online resource 3. Therefore, it is assumed that even with a small number of selected RMCE subclones, different cell lines with highly similar transcription efficiencies, indicated by the specific RNA/DNA ratio, can be obtained. By using conventional random transgene integration much higher screening effort is necessary to find two subclones with similar expression behavior. This is because random integration of transgenes into different chromosomal loci results in different transcription efficiencies and additional positional effects contribute to the final mRNA levels together with different RNA polymerase/DNA interactions.

**Fig. 3:**
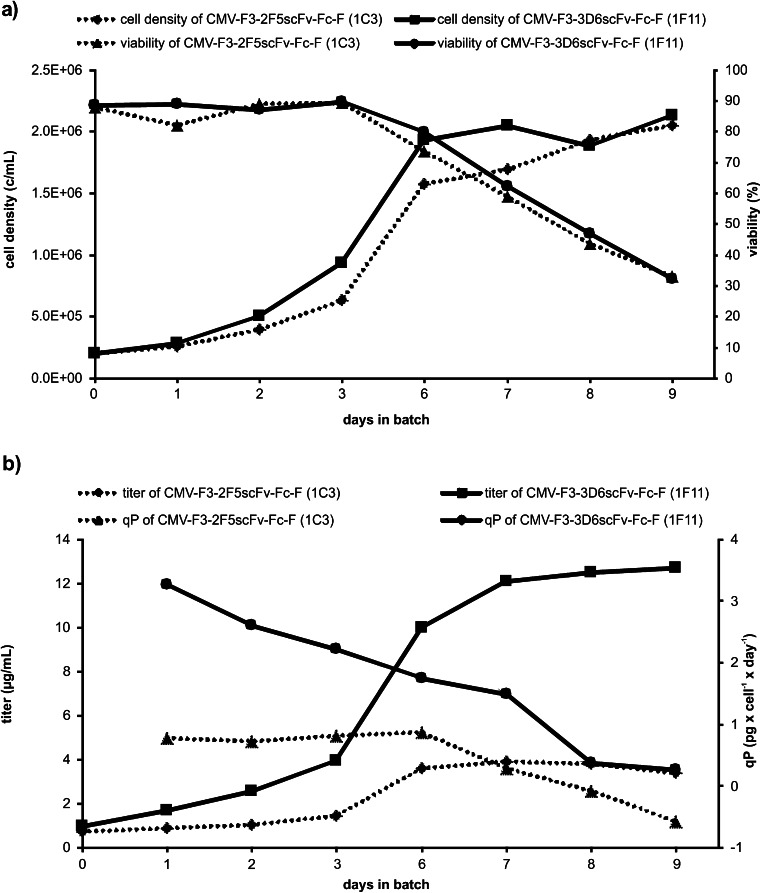
Batch experiments of subclone CMV-F3-3D6scFv-Fc-F 1F11 and CMV-F3-2F5scFv-Fc-F 1C3 in spinner flasks. 1F11 and 1C3 were selected for spinner batch experiments by seeding 2 × 10^5^ cells/mL in ProCHO5 supplemented with 4 mM l-glutamine and hypoxanthine/thymidine without using any selection pressure. During the 9-day batch experiment, cell density and viability (**a**) as well as specific productivity qp and final product titer (**b**) were measured

Although RMCE exchange gave highly similar productivities and growth rates (Fig. 2a, b), little variance can be observed due to quantitation variance or local heterogeneity in culture conditions such as temperature or nutrient gradients during T25 flask cultivation. Therefore, the two best performing 3D6scFv-Fc (1F11 and 3B9) and 2F5scFv-Fc (1C3 and 3E5) clones were selected based on qp and μ for consecutive passages in spinner vessels for 40 days without any selection pressure. During this period of routine cultivation, similar specific growth rates were observed for all scFv-Fc-producing subclones (Fig. 2c). A factor two difference was noted for the specific productivities of 3D6scFv-Fc-producing subclones compared with 2F5scFv-Fc-producing subclones (Fig. 2d). In the batch experiments without selection pressure, the maximum cell density reached 2 × 10^6^ cells/mL after 7 or 9 days for subclone CMV-F3-3D6scFv-Fc-F 1F11 or CMV-F3-2F5scFv-Fc-F 1C3, respectively (Fig. 3a). Whereas maximum cell viability could be maintained for 3 days, it dropped to 30 % after 9 days in batch culture for both cell clones. A final product titer of 13 μg/mL was obtained for subclone CMV-F3-3D6scFv-Fc-F 1F11 exhibiting a constantly decreasing specific productivity starting from 3.3 pg cell^−1^ day^−1^. For subclone CMV-F3-2F5scFv-Fc-F 1C3, a final titer of only 3.4 μg/mL could be reached with a specific productivity of 0.8 pg cell^−1^ day^−1^ that remained constant for the first 6 days during batch cultivation.

### Intracellular product formation and mRNA levels of scFv-Fc-producing subclones

**Fig. 4:**
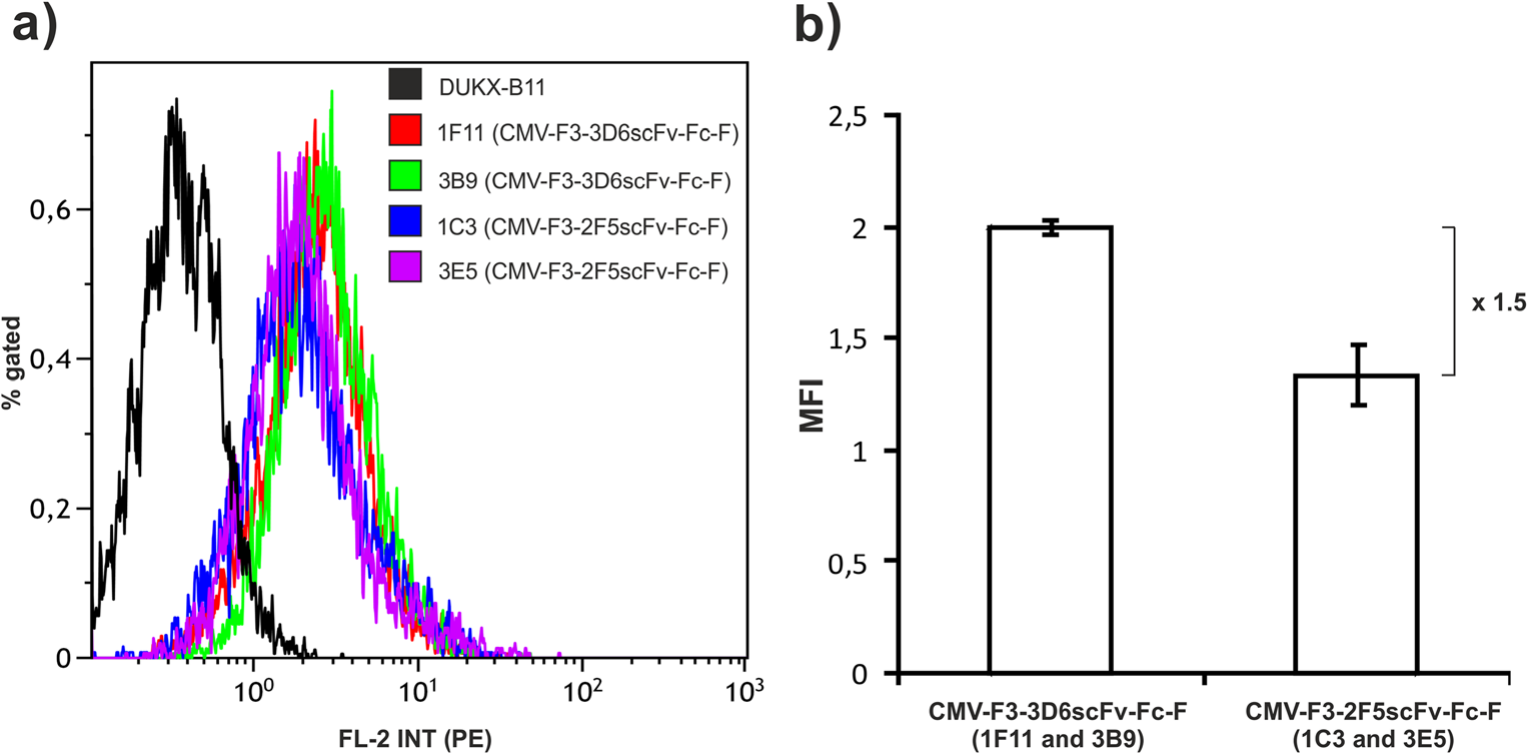
Flow cytometric analysis of intracellular product formation of four selected scFv-Fc clones cultivated in spinner flasks. Samples were taken at three different days from four scFv-Fc-producing cell clones cultivated in spinner flasks. Cells were fixed by ethanol treatment and labeled with anti-huIgG-γ-chain-R-phycoerythrin (PE) antibody. **a** Single-parameter FL-2 histogram of samples taken at day 40 in spinner cultivation. DUKX-B11 host cell line was used as negative control. **b** Mean values of intracellular product formation of two 3D6scFv-Fc- or 2F5scFv-Fc-producing cell clones for each antibody variant are compared by median fluorescence intensities (MFI) showing a difference of 1.5 in intracellular product content. *Error bars* represent ±SEM

**Fig. 5:**
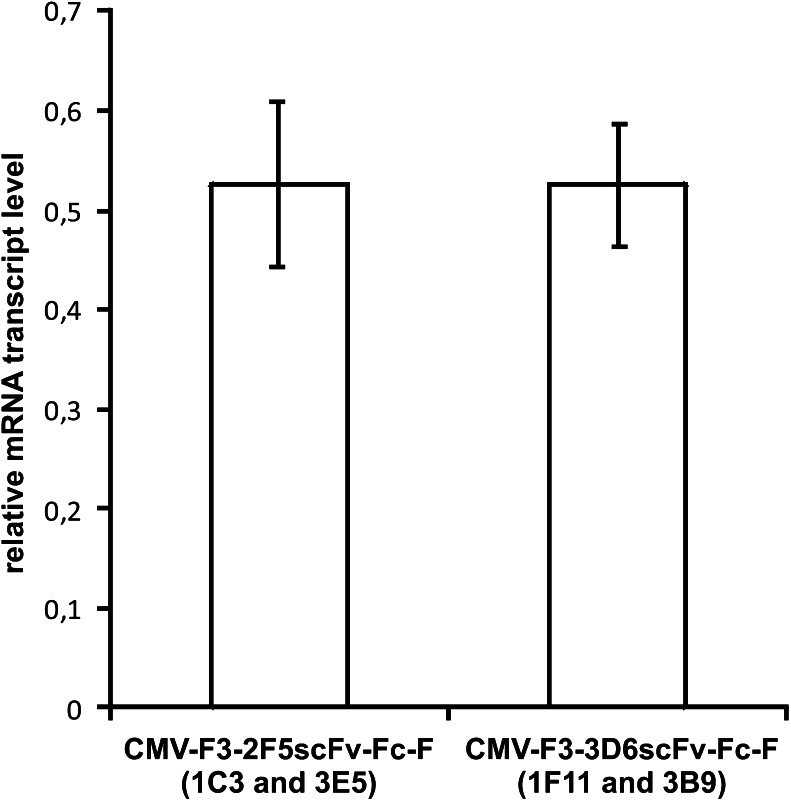
qPCR analysis of mRNA transcript level of two selected 3D6scFv-Fc- or 2F5scFv-Fc-producing RMCE clones cultivated in spinner flasks. Samples were taken at three different days and measured in two technical replicates. Total mRNA was reverse transcribed into cDNA and analyzed by qPCR using probes specific for the Fc sequence or β-actin used as an internal standard. Mean 2^−ΔCp^ values were calculated based on differences of Cp values between β-actin and the Fc sequence. *Error bars* represent standard deviation

The four scFv-Fc-producing cell clones 1F11, 3B9, 1C3, and 3E5 chosen for routine cultivation in spinner flasks were analyzed for intracellular product formation and mRNA levels at three independent sampling days (indicated by arrows in Fig. 2c, d) to identify possible bottlenecks responsible for the differences in specific productivities of the two antibody variants. A single-parameter histogram at day 40 in spinner cultivation is depicted in Fig. 4a. A single peak of intracellular PE fluorescence (FL-2 signal intensity) was obtained from intracellular scFv-Fc for all individual sampling days (Online resource 4), as was expected for a RMCE guided exchange of transgenes. Comparing the median fluorescence intensity of two 2F5scFv-Fc and 3D6scFv-Fc-producing cell clones only minor variations were measured between the different sampling times indicated by small error bars. The median fluorescence intensity (MFI) of 3D6scFv-Fc-producing clones was a factor 1.5 higher than the 2F5scFv-Fc-producing clones (Fig. 4b). This difference does not necessarily reflect the 2-fold difference in specific productivities. Relative mRNA levels compared with beta-actin reference level were constant in all scFv-Fc-producing RMCE clones cultivated in spinner flasks (Fig. 5; Online resource 5). These data support the capacity of the new host cell line as a tool for the comparison of clones at a post transcriptional level.

## Discussion

Genomic targets enabling FLP RMCE can either be established by random integration (electroporation and transfection; Turan et al. 2013) or using the scout functions and integration preferences of retroviral vectors (Kuehle et al. 2014). Once the single-copy status has been documented the respective candidate sites lend themselves to the FLP-mediated integration of compatible expression cassettes. These conversions maintain the single-copy status of the target, they do not introduce unwanted vector sequences nor do they cause inadvertent rearrangements due to the underlying enzymatic principles.

In biotechnology, common exploitations of this strategy concern the definition of unique highly expressed loci for which a comprehensive toolbox has been introduced by Qiao et al. (2009). Based on the available target and donor vector backbones, our present study follows somewhat different goals, as it addresses the expression potential of various transgene architectures. This approach is valid if candidate constructs can be compared at predefined sites with moderate or even low intrinsic expression potential. Access to these requirements can be provided in case the general selection scheme (illustrated in Fig. 1) is streamlined in that only the second round of selections (step 3) is stringently controlled whereas step 2 obeys relaxed conditions that can be verified with just moderate investments of time.

The aim of this study was to establish the DUKX-B11 F3/F host cell line capable of introducing different antibody variants into the same genomic locus by RMCE. The primary goal was to develop this target site for a controlled and predictable integration of different genes of interest into the same chromosomal position irrespective of the achieved final specific productivity. Therefore, in this study no effort was made to identify high-producer clones by stringent and time-consuming screening and selection procedures or to characterize the targeted genomic locus in more detail by southern blot analysis or DNA sequencing methods. The rationale behind the development of the RMCE host cell line DUKX-B11 F3/F is to provide a tool that enables investigation of the expression levels of different proteins, in particular antibody variants, in an industrially widely used cell line under reproducible and identical transcriptional status of the integrated gene cassettes. Therefore, the primary focus was given to select RMCE compatible target sites with long-term stability and accessibility to the transcription machinery for gene expression as well as accessibility to the FLP recombinase enzyme to ensure efficient RMCE reactions.

The initial RMCE-competent target cassette, pCMV-F3-gfp-F, is controlled by a CMV promoter upstream of the *FRT*3 site resulting in a promoter trap as described in Qiao et al. (2009) (Fig. 1, step 1). The promoter trap overcomes expression of randomly introduced transgenes not targeted specifically to the chromosomal RMCE target site by FLP mediated recombination. The procedure for establishing DUKX-B11 F3/F was based on the expression of the CMV-F3-gfp-F cassette without selection for stable gfp-producing cell clones in a way that potent RMCE targets were already available 24 h after the first transfection with pCMV-F3-gfp-F. The first RMCE recombination events between the target cassette CMV-F3-gfp-F and the donor cassette F3-hyg/tk-F might have occurred either at the chromosomal level, where the gfp cassette was integrated randomly into the genome and afterwards the replacement occurred by RMCE. Alternatively and also possible, the cassette exchange reactions might have occurred already at the transient episomal stage where RMCE was initiated between the two cassettes still present as episomal plasmids. However, either way, the gfp cassette needs to be replaced by the hyg/tk fusion cassette by RMCE and integrated into the host genome leading to hyg/tk expression driven by the CMV promoter introduced by the first parental plasmid. The stability and accessibility of the donor cassette CMV-F3-hyg/tk-F could be demonstrated by cultivation in hygromycin B, followed by induction of a second RMCE reaction to replace the F3-hyg/tk-F cassette by another RMCE donor cassette, F3-gfp/tk/neo-F (step 3 in Fig. 1) followed by G418 selection. This second RMCE reaction and positive selection procedure is necessary to establish cell clones with high RMCE potential, at loci with appropriate transcription (mRNA) levels. At this point a negative selection procedure would be possible by using ganciclovir to select clones that have lost the tk fusion protein by site-specific cassette exchange.

The individual RMCE reactions were tracked with specific PCR reactions (Table 1; Online resource 1), indicating the success of both cassette exchange events. Absence of specific amplicons for hyg and presence of amplicons specific for the neo sequence under control of the CMV promoter in the final RMCE host DUKX-B11 F3/F indicated a successful integration of the triple fusion protein gfp/tk/neo into the target site by RMCE. The final RMCE host cell line DUKX-B11 F3/F resists G418 and exhibits a gfp-positive phenotype as indicated by flow cytometry (single-parameter FL-1 INT histogram in Fig. 1). The homogenous gfp signal profile, indicated by similar median, mean and mode values, implies that gfp expression in all cells of the population is driven by the same transcription control elements as was expected for a targeted RMCE integration of the gfp cassette. FISH results indicate a single integration locus of the gfp/tk/neo RMCE-targeting cassette in DUKX-B11 F3/F (Online resource 2).

For proof of concept, 3D6scFv-Fc and 2F5scFv-Fc antibody variants were stably introduced into DUKX-B11 F3/F employing the tagged RMCE locus (Fig. 2; Online resource 3). The analysis of twelve subclones of each variant showed similar growth characteristics but protein specific differences in specific secretion levels (Fig. 2a, b). Importantly, the two selected scFv-Fc producing subclones for each variant proved stable and highly similar specific productivities as indicated by small error bars in Fig. 2d. This demonstrates the success of targeted integration of different transgenes by RMCE to induce predictable and reproducible expression levels in DUKX-B11 F3/F. The capability of DUKX-B11 F3/F for targeted integration of different genes of interest into RMCE target sites for reproducible expression patterns is supported by homogenous and similar intracellular scFv-Fc expression patterns (Fig. 4), as well as reproducible and stable transgene mRNA levels (Fig. 5). Thereby, DUKX-B11 F3/F is suitable to investigate product specific differences in the posttranscriptional expression cascade under a chromatin status-independent setup.

A factor of 2- to 3-fold higher specific productivity could be detected for 3D6scFv-Fc subclones compared with 2F5scFv-Fc subclones using DUKX-B11 F3/F as the expression host for targeted integration of the transgenes by RMCE. The pronounced differences in specific productivity were observable during continuous T25 and spinner flask cultivation as well as in batch culture experiments. Mader et al. (2013) reported a factor of three to 4-fold lower specific productivity of 2F5scFv-Fc-producing subclones compared with 3D6scFv-Fc cell clones established by random introduction of conventional plasmid expression vectors or alternatively by bacterial artificial chromosomes (BAC) harboring the same expression elements.

The four step procedure to generate antibody producing DUKX-B11 clones presented in this study has certain advantages compared with simple two-step “tag-and-exchange” strategies reported in literature (Askew et al. 1993). The introduction of a first gfp cassette under control of a trapped promoter allows the easy verification of transfection efficiency in the transient stage. At this point also, a selection of gfp high-producing cells could be performed by fluorescence-activated cell sorting as was demonstrated in Qiao et al. (2009). It is known that the efficiency of RMCE is dependent on the chromosomal position of the integrated target cassette as was demonstrated by Vooijs et al. (2001) for the Cre/loxP system. CHO cells are especially prone to spontaneous genetic rearrangements (Cao et al. 2012; Kim et al. 2011) that might lead to a loss of RMCE capability of the targeted chromosomal cassette. To ensure a RMCE-competent locus, the stably introduced hyg/tk cassette was exchanged by two additional RMCE reactions to introduce the gfp/tk/neo fusion and finally the scFv-Fc antibody cassette. The finally generated scFv-Fc producer cell lines demonstrate the long-term potency of the target site for RMCE by two different transgene cassettes with varying nucleotide sequence length between the two heterospecific *FRT* sites.

Although the primary goal of this study was not to generate a RMCE host cell line conferring high specific productivities, if desired, improvements in qP can be reached by systematic use of S/MAR elements (Qiao et al. 2009). An alternative option is enabled by lentiviral transduction, at the single-copy level into transcriptionally competent loci as described by Oberbek et al. (2011). The system could also be improved by using bacterial artificial chromosomes (BAC) as open chromatin and copy number-dependent expression vehicles (Blaas et al. 2009) in combination with serial, accumulative RMCE reactions for introduction of multiple gene copies with various available heterospecific *FRT* sites into the same genomic locus. We wish to emphasize, however, that for present purposes, a less stringent selection procedure for choosing chromosomal loci with moderate expression capabilities was used to investigate transgene-intrinsic limitations in low-producing antibody variants (exemplified by 2F5scFv-Fc) that might otherwise be overlaid by the high-expression activity of a high-expression locus.

Global analysis methods in systems biology to identify bottlenecks common to different antibody producer cell clones attract more and more attention (Gupta and Lee 2007). One limitation in different -omics techniques is the generation of subclones with identical transcription capabilities derived from the same transfection reaction or, even more challenging, derived from different transfection reactions of different antibody variants. The difficulties arise on the one hand from randomly introduced expression cassettes leading to unpredictable gene copy numbers and different transcription efficiencies influenced by different surrounding chromosomal environments (position effect). We here propose that the new available DUKX-B11 F3/F cell line is a RMCE-competent host to facilitate the rapid generation of subclones with comparable and stable gene copy numbers, mRNA, and intracellular product levels guided by integration into the preselected chromosomal locus. Proof of concept was provided during the host cell development with the exchange of the initial cassette for the active heterologous transgene cassette (CMV-F3-gfp/tk/neo-pA-F). Therefore, it is expected that exchange of this cassette with any scFv-Fc coding sequence will lead to clones that are more robust against epigenetic silencing and genetic instabilities compared with subclones generated by transgene integration into “new and unknown” chromosomal sites by random integration events. It can be expected that the DUKX-B11 F3/F host cell line is useful in systems biology in general and in following proteomics studies, as an explicit application, to establish different antibody producing cell lines with moderate but highly similar and robust expression capabilities more rapidly since the adaption process is shortened by targeted integration into an active and stable locus.

Further analysis of the generated scFv-Fc-producing cell clones by qPCR analysis with primers specific for targets other than β-actin might be used for a more in-depth comparison of the generated scFv-Fc-producing subclones. Such targets might include components of the ER compartment, apoptotic pathway, or folding/secretion machinery.

In summary, a stable RMCE host, DUKX-B11 F3/F, could be established with homogenous expression capabilities of a gtn fusion protein. The stably integrated RMCE target sites can be replaced by different genes of interest leading to homogenous cell populations. We were able to develop recombinant clones with stable and reproducible mRNA levels and intracellular product formation, as well as similar growth and expression capabilities controlled by targeted integration of transgene cassettes into the same chromosomal environment by RMCE.

Our study significantly expands existing RMCE tools by a slight modification of already existing strategies to generate genomic target loci to guide different transgenes specifically into chromosomal loci with reproducible transcription characteristics.

## Electronic supplementary material

Below is the link to the electronic supplementary material.

ESM 1 (PDF 475 kb)

## References

[CR1] Aggarwal SR (2014). What’s fueling the biotech engine—2012 to 2013. Nat Biotechnol.

[CR2] Askew GR, Doetschman T, Lingrel JB (1993). Site-directed point mutations in embryonic stem cells: a gene-targeting tag-and-exchange strategy. Mol Cell Biol.

[CR3] Blaas L, Musteanu M, Eferl R, Bauer A, Casanova E (2009). Bacterial artificial chromosomes improve recombinant protein production in mammalian cells. BMC Biotechnol.

[CR4] Cao Y, Kimura S, Itoi T, Honda K, Ohtake H, Omasa T (2012). Construction of BAC-based physical map and analysis of chromosome rearrangement in Chinese hamster ovary cell lines. Biotechnol Bioeng.

[CR5] Clarke C, Doolan P, Barron N, Meleady P, O’Sullivan F, Gammell P, Melville M, Leonard M, Clynes M (2011). Predicting cell-specific productivity from CHO gene expression. J Biotechnol.

[CR6] Datta P, Linhardt RJ, Sharfstein ST (2013). An 'omics approach towards CHO cell engineering. Biotechnol Bioeng.

[CR7] Dietmair S, Nielsen LK, Timmins NE (2012). Mammalian cells as biopharmaceutical production hosts in the age of omics. Biotechnol J.

[CR8] Doolan P, Clarke C, Kinsella P, Breen L, Meleady P, Leonard M, Zhang L, Clynes M, Aherne ST, Barron N (2013). Transcriptomic analysis of clonal growth rate variation during CHO cell line development. J Biotechnol.

[CR9] Fukushige S, Sauer B (1992). Genomic targeting with a positive-selection lox integration vector allows highly reproducible gene expression in mammalian cells. Proc Natl Acad Sci U S A.

[CR10] Gupta P, Lee KH (2007). Genomics and proteomics in process development: opportunities and challenges. Trends Biotechnol.

[CR11] Huang Y, Li Y, Wang YG, Gu X, Wang Y, Shen BF (2007). An efficient and targeted gene integration system for high-level antibody expression. J Immunol Methods.

[CR12] Kantardjieff A, Nissom PM, Chuah SH, Yusufi F, Jacob NM, Mulukutla BC, Yap M, Hu WS (2009). Developing genomic platforms for Chinese hamster ovary cells. Biotechnol Adv.

[CR13] Kim M, O’Callaghan PM, Droms KA, James DC (2011). A mechanistic understanding of production instability in CHO cell lines expressing recombinant monoclonal antibodies. Biotechnol Bioeng.

[CR14] Kim JY, Kim YG, Lee GM (2012). CHO cells in biotechnology for production of recombinant proteins: current state and further potential. Appl Microbiol Biotechnol.

[CR15] Kito M, Itami S, Fukano Y, Yamana K, Shibui T (2002). Construction of engineered CHO strains for high-level production of recombinant proteins. Appl Microbiol Biotechnol.

[CR16] Kuehle J, Turan S, Cantz T, Hoffmann D, Suerth JD, Maetzig T, Zychlinsk D, Klein C, Steinemann D, Baum C, Bode J, Schambach A (2014) Modified lentiviral LTRs allow Flp recombinase-mediated cassette exchange and in vivo tracing of „factor-free“ induced pluripotent stem cells. Mol Ther 22(5):919–28. doi:10.1038/mt.2014.4 10.1038/mt.2014.4 PMC4015239 24434935

[CR17] Kwaks TH, Otte AP (2006). Employing epigenetics to augment the expression of therapeutic proteins in mammalian cells. Trends Biotechnol.

[CR18] Lattenmayer C, Trummer E, Schriebl K, Vorauer-Uhl K, Mueller D, Katinger H, Kunert R (2007). Characterisation of recombinant CHO cell lines by investigation of protein productivities and genetic parameters. J Biotechnol.

[CR19] Livak KJ, Schmittgen TD (2001). Analysis of relative gene expression data using real-time quantitative PCR and the 2(-Delta Delta C(T)) Method. Methods.

[CR20] Mader A, Prewein B, Zboray K, Casanova E, Kunert R (2013). Exploration of BAC versus plasmid expression vectors in recombinant CHO cells. Appl Microbiol Biotechnol.

[CR21] Mutskov V, Felsenfeld G (2004). Silencing of transgene transcription precedes methylation of promoter DNA and histone H3 lysine 9. EMBO J.

[CR22] Nehlsen K, Schucht R, da Gama-Norton L, Krömer W, Baer A, Cayli A, Hauser H, Wirth D (2009). Recombinant protein expression by targeting pre-selected chromosomal loci. BMC Biotechnol.

[CR23] Oberbek A, Matasci M, Hacker DL, Wurm FM (2011). Generation of stable, high-producing CHO cell lines by lentiviral vector-mediated gene transfer in serum-free suspension culture. Biotechnol Bioeng.

[CR24] O’Gorman S, Fox DT, Wahl GM (1991). Recombinase-mediated gene activation and site-specific integration in mammalian cells. Science.

[CR25] Qiao J, Oumard A, Wegloehner W, Bode J (2009). Novel tag-and-exchange (RMCE) strategies generate master cell clones with predictable and stable transgene expression properties. J Mol Biol.

[CR26] Raymond CS, Soriano P (2007). High-efficiency FLP and PhiC31 site-specific recombination in mammalian cells. PLoS One.

[CR27] Richards EJ, Elgin SC (2002). Epigenetic codes for heterochromatin formation and silencing: rounding up the usual suspects. Cell.

[CR28] Sommeregger W, Prewein B, Reinhart D, Mader A, Kunert R (2013). Transgene copy number comparison in recombinant mammalian cell lines: critical reflection of quantitative real-time PCR evaluation. Cytotechnology.

[CR29] Turan S, Zehe C, Kuehle J, Qiao J, Bode J (2013). Recombinase-mediated cassette exchange (RMCE)—a rapidly-expanding toolbox for targeted genomic modifications. Gene.

[CR30] Untergasser A, Nijveen H, Rao X, Bisseling T, Geurts R, Leunissen JA (2007). Primer3Plus, an enhanced web interface to Primer3. Nucleic Acids Res.

[CR31] Urlaub G, Chasin L (1980) Isolation of Chinese hamster cell mutants deficient in dihydrofolate reductase activity. Proc Natl Acad Sci U S A 77:4216–4220 10.1073/pnas.77.7.4216 PMC349802 6933469

[CR32] Vooijs M, Jonkers J, Berns A (2001). A highly efficient ligand-regulated Cre recombinase mouse line shows that LoxP recombination is position dependent. EMBO Rep.

[CR33] Wilson C, Bellen HJ, Gehring WJ (1990). Position effects on eukaryotic gene expression. Annu Rev Cell Biol.

[CR34] Wurm FM (2004). Production of recombinant protein therapeutics in cultivated mammalian cells. Nat Biotechnol.

