# Supplementary material for: Accurate comparison of antibody expression levels by reproducible transgene targeting in engineered recombination-competent CHO cells
Source: Appl Microbiol Biotechnol. 2014 Aug 27;98(23):9723–33. doi: 10.1007/s00253-014-6011-1 (PMC4231286; doi:10.1007/s00253-014-6011-1)
Supplement: Supplementary file 1 — (PDF 475 kb) [file 253_2014_6011_MOESM1_ESM.pdf]

Supplementary Material

Applied Microbiology and Biotechnology

Accurate comparison of antibody expression levels by reproducible transgene targeting in engineered recombination-competent CHO cells

Mayrhofer P.[1], Kratzer B.[2], Sommeregger W.[1], Steinfellner W.[1], Reinhart D.[1], Mader A.[1], Turan S.[3], Qiao J.[4], Bode J.[5] , Kunert R.[1]*

[1] Department of Biotechnology/Vienna Institute of BioTechnology (BOKU – VIBT), University of Natural Resources and Life Sciences (Vienna), Muthgasse 18, A-1190 Vienna, Austria

[2] Institute of Immunology, Medical University Vienna, Borschkegasse 8a, A-1090 Vienna, Austria

[3] Department of Genetics, Stanford University School of Medicine, Alway Building, Room M-334, 300 Pasteur Drive, Stanford, CA 94305-5120, USA

[4] WuXi App Tec Co. Ltd, 288 Fute Zhong Road, Shanghai 200131, China

[5] Hannover Medical School/Institute for Experimental Hematology, OE 6960, Carl-Neuberg-Str.1, D-30625 Hannover, Germany

* Corresponding author: email: renate.kunert@boku.ac.at; tel: +43 1 47654 6595; fax: +43 1 47654 6675

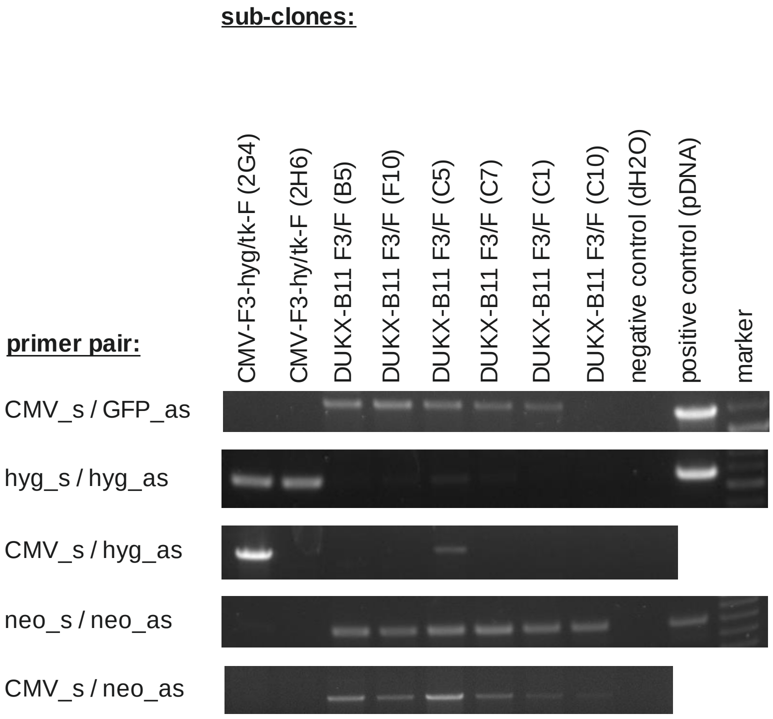

Online Resource 1 PCR characterization of genomic DNA isolated from different CMV-F3-hyg/tk-F or DUKX-B11 F3/F subclones as summarized in Table 1.

Subclone CMV-F3-hyg/tk-F (2G4) showed an authentic RMCE reaction with the first RMCE donor cassette (step 2 in Fig. 1) and was selected for the second RMCE reaction resulting in the final DUKX-B11 F3/F (B5) clone showing the gfp/tk/neo (GTN) cassette integrated behind the CMV promoter by RMCE. If available, plasmid DNA (pDNA) was used as positive control

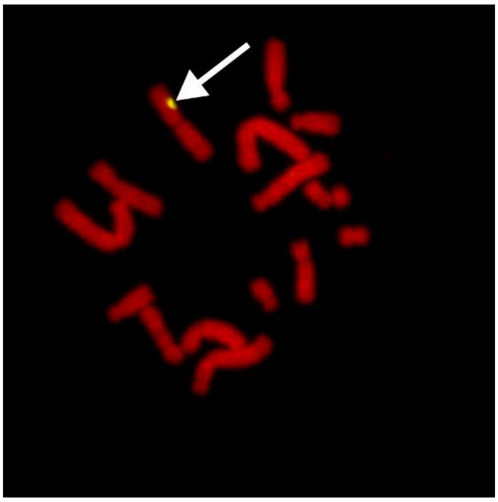

Online Resource 2 Fluorescence in-situ hybridization (FISH) analysis of DUKX-B11 F3/F with probes specifically binding to the CMV-F3-gfp/tk/neo-F RMCE target site.

3 µg of plasmid DNA pF3-gfp/tk/neo-F was labeled using the DIG-Klenow-labeling kit DIG High prime (Roche) according to the manufacturer's protocol. For preparation of metaphase spreads cells in exponential phase were treated with 0.2 µg/mL demecolcine for 4 hours. DIG-labeled and hybridized probes were detected using triple detection antibody setup. Sequential addition of mouse anti-DIG (Roche), goat anti-mouse-FITC and rabbit anti-goat-FITC detection antibodies gave a single fluorescent locus (white arrow) under a SP5 II laser scanning confocal microscope (Leica) at 488 nm using propidium iodide counter-staining at 561 nm

a.)

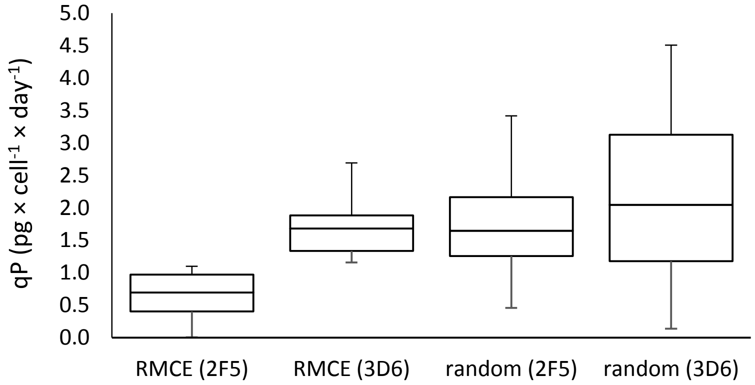

b.)

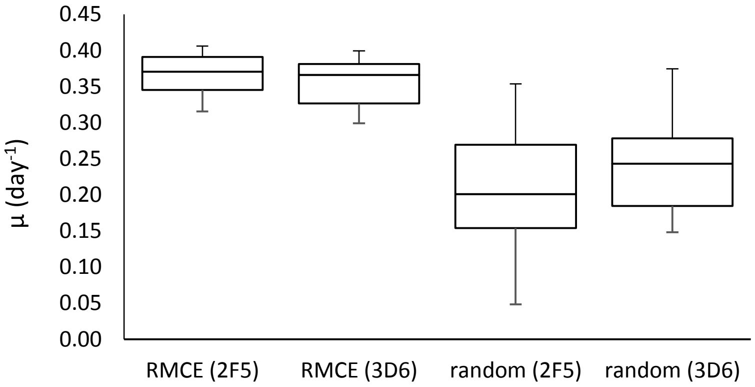

Online Resource 3 Distribution of specific productivities and growth rates of scFv-Fc producing subclones developed by targeted RMCE compared to random integration of the transgene.

RMCE subclones were developed as described in Materials and Methods and analyzed as described in Fig. 2a and Fig. 2b. Subclones generated by random integration of the plasmid DNA were established by using the same transfection and selection procedures as with the RMCE subclones. (**A**) Specific productivities (qP) with an interquartile range (midspread) of 0.56 and 0.54 pg × cell^{-1} × day^{-1} of the 2F5scFv-Fc and 3D6scFv-Fc subclones generated by RMCE or 0.91 and 1.95 pg × cell^{-1} × day^{-1} of 2F5scFv-Fc and 3D6scFv-Fc generated by random integration of the transgenes, respectively. (**B**) Specific growth rates (µ) with an interquartile range (midspread) of 0.05 and 0.05 day^{-1} of the 2F5scFv-Fc and 3D6scFv-Fc subclones generated by RMCE or 0.12 and 0.09 day^{-1} of 2F5scFv-Fc and 3D6scFv-Fc generated by random integration of the transgenes, respectively

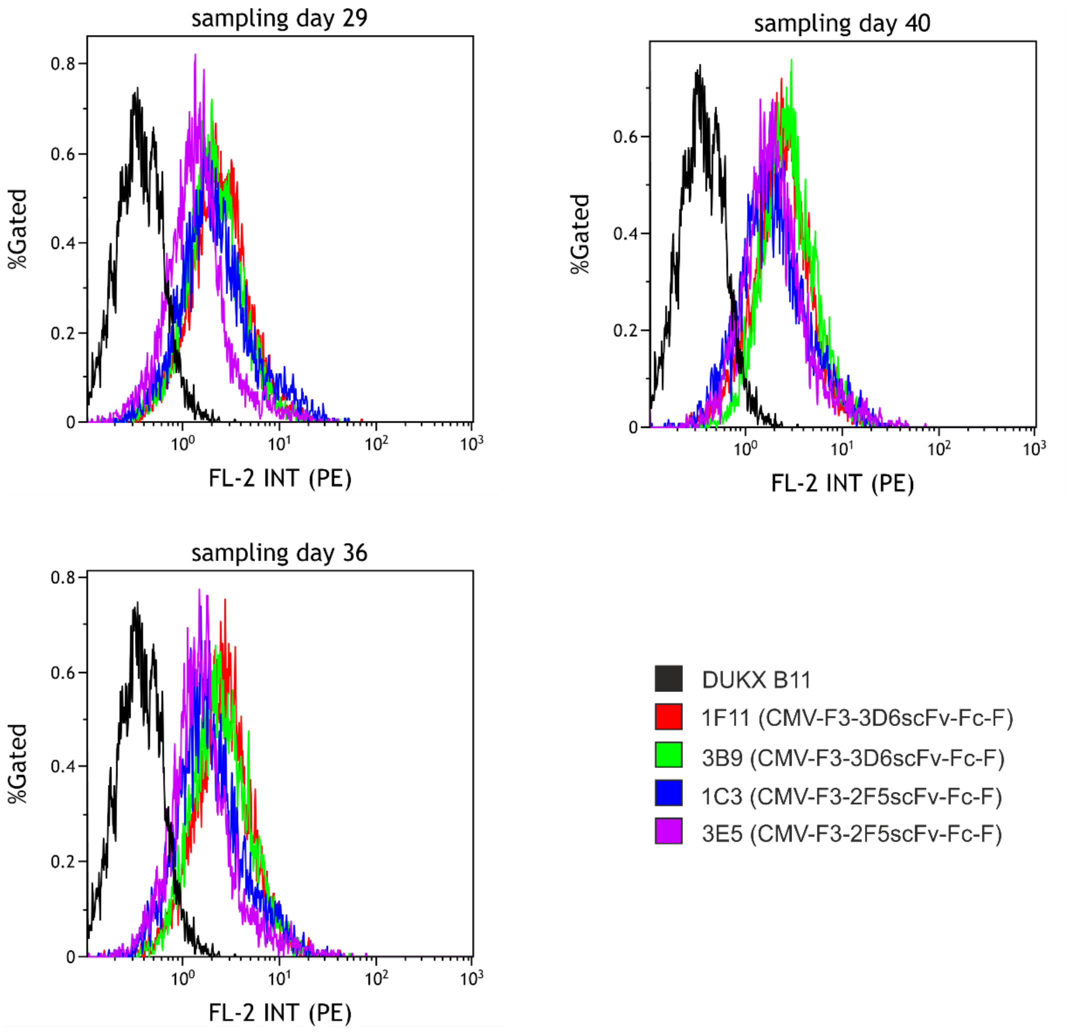

Online Resource 4 Homogenous intracellular product formation of scFv-Fc producing subclones at three independent sampling days measured by flow cytometry.

ScFv-Fc producing subclones cultured in spinner flasks were fixed by ice-cold ethanol and labeled with anti-huIgG-γ-chain-R-phycoerythrin (PE) antibody. Single parameter FL-2 histograms were used to determine the mean values of the median fluorescence intensities (MFI) at three independent sampling days, as indicated in Fig. 4b

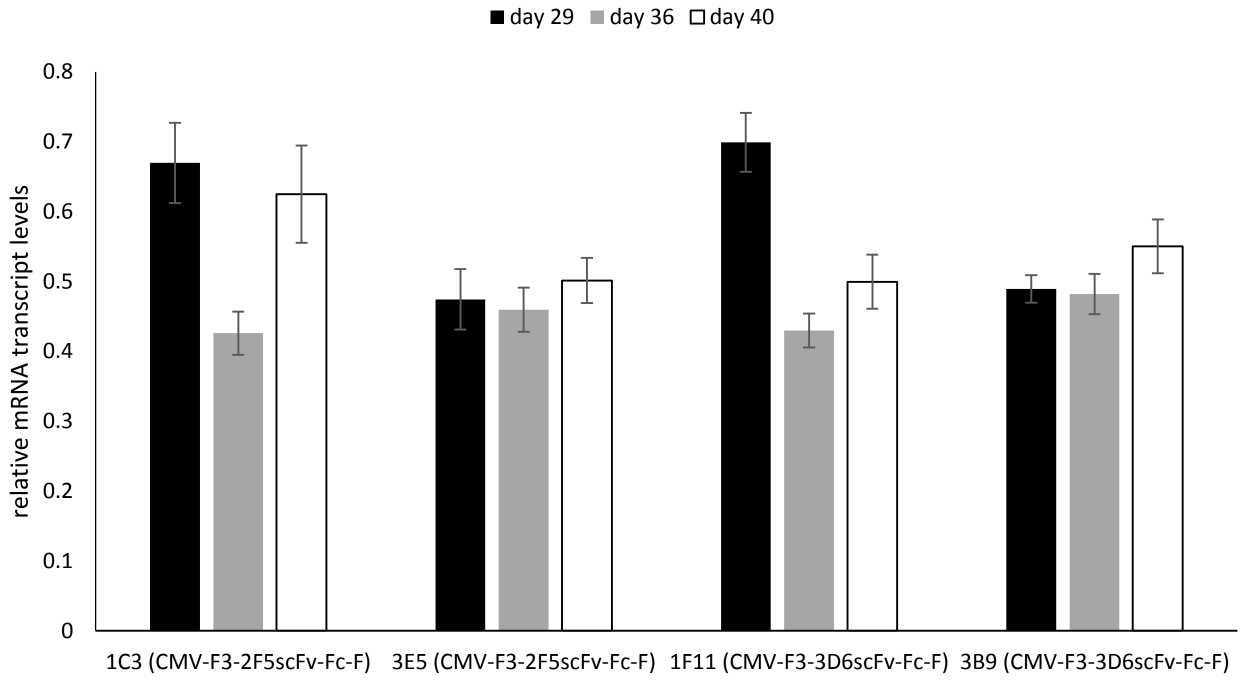

Online Resource 5 Relative mRNA transcript levels of two 2F5scFv-Fc producing subclones and two 3D6scFv-Fc producing subclones sampled at three independent culture days.

Samples were measured in two technical and three biological replicates. Total mRNA was reverse transcribed into cDNA and analyzed by qPCR using probes specific for the Fc sequence or β-actin used as an internal standard. Mean $2^{-\Delta Cp}$ values were calculated based on differences of Cp values between β-actin and the Fc sequence. Error bars represent standard deviation
